# Commentary: Estimands in cluster trials: thinking carefully about the target of inferenceand the consequences for analysis choice

**DOI:** 10.1093/ije/dyac174

**Published:** 2022-08-26

**Authors:** Karla Hemming, Monica Taljaard

**Affiliations:** Institute of Applied Health Research, The University of Birmingham, Birmingham, UK; Clinical Epidemiology Program, Ottawa Hospital Research Institute, Ottawa, Ontario, Canada; School of Epidemiology, Public Health and Preventive Medicine, University of Ottawa, Ottawa, Canada

## Specifying the target of inference in cluster trials

Cluster randomized trials (CRTs) are complex.^1^ At the protocol development stage, we have to select an appropriate unit of randomization (which may depend on the unit of intervention delivery) and an appropriate unit of analysis (which may depend on the unit of observation).^2^^,^^3^ If the unit of randomization is different from the unit of analysis, we must account for clustering among multiple observations from the same cluster—a requirement that is well appreciated.^2^ The unit of analysis may be either the individual or the cluster, with the choice ideally made on statistical grounds (although in practice it may reflect personal preference, convenience or experience).^3^ The paper by Kahan and colleagues advises us that we also need to choose an a priori unit of inference and this choice is critical in selecting both the unit and the method of analysis.^4^ We believe that the need to consider the target of inference before specifying the method of analysis has not received adequate attention in the cluster trials literature to date.

Defining the unit of inference, i.e. the estimand of interest, is essentially about carefully specifying the research question.^5^^,^^6^ Specifically, we must consider whether interest lies in determining:

- the effect of the intervention on a typical individual, or
- the effect of the intervention on a typical cluster.

It is important to realize that the estimated treatment effect for these two questions can differ in the same trial for the same outcome. In particular, the treatment effect will differ when cluster sizes are informative—which essentially means either the outcomes vary across clusters depending on cluster size and/or the treatment effect varies across clusters depending on cluster size (i.e. a cluster size by treatment interaction).^7^^,^^8^ Informative cluster sizes are not expected to be uncommon in practice. The precise circumstances under which these treatment effects differ depend on the type of treatment effect (i.e. the measure of association). For mean difference, risk difference or relative risk, differences only arise if there is an interaction between the treatment effect and cluster size; for odds ratios and hazard ratios, the differences arise even if there is no treatment by cluster size interaction, but the baseline prevalence varies across cluster sizes.

## Choosing an analytical strategy to match the unit of inference

It is also important to realize that choosing the desired unit of inference is distinct from choosing the unit of analysis: regardless of whether the effect on a typical individual or typical cluster is of interest, it is possible to conduct either an individual-level analysis or a cluster-level analysis.^4^ However, exactly how to carry out these analyses to ensure they answer the question of interest requires careful consideration. In the case of an individual-level analysis, two commonly used methods are the generalized linear mixed model (GLMM) or generalized estimating equations (GEEs).^3^^,^^9^ Whilst these individual-level methods of analysis have advantages, both GLMM and GEE models can produce biased estimates when cluster sizes are informative.^4^^,^^7^ Conversely, a cluster-level analysis can yield an unbiased estimate for the effect on the typical cluster even when cluster sizes are informative; however, if we choose a weighting method to preserve statistical efficiency, we can inadvertently introduce a bias.^4^

A cluster-level analysis is probably the least-commonly used approach in practice, even though it generally produces valid inferences.^9^ When clusters vary in size, clusters may be weighted to improve efficiency.^10^ However, Kahan *et al*. show that the question of when a cluster-level analysis should be weighted turns out to be subtler. In fact, a cluster-level approach, without any weighting of cluster sizes, even when cluster sizes vary, will allow estimation of the impact of the intervention for the average cluster. Weighting by cluster sizes, i.e. by the number of individuals within that cluster, changes the target of inference to the average individual. Thus, the question of whether a cluster-level analysis should be weighted is not first a question of a gain in statistical efficiency, but rather about whether the objective is to estimate the impact of the intervention for the average cluster or the average individual.

Kahan *et al.* identify what might be considered a more surprising result. Suppose it is of interest to estimate the impact of the intervention on the average individual. Here, a common approach is to use a GLMM or GEEs assuming a working exchangeable correlation structure.^3^ It transpires that both of these approaches do not target the effect for the average individual when cluster size is informative. This means that whenever a generalized estimating equation (with exchangeable correlation structure) or mixed model is used to evaluate the impact of the intervention for the average individual, this will yield a biased estimate of the effect (again when cluster size is informative). The recommended approach is to use GEEs assuming an independent working correlation structure or ordinary regression with cluster-robust standard errors.^4^

## Other issues to consider when specifying the target of inference

Thinking carefully about the target of inference is not a new concept.^9^^,^^11^ There are of course other factors to consider when thinking about the target of inference and choosing the method of analysis in cluster trials—notably whether interest is in the marginal (typically obtained via GEEs, but can be obtained via GLMMs) or cluster-specific effects (obtained via GLMMs).^12^ The marginal effect allows us to consider the effect of the intervention for a population of clusters and individuals similar to those included in the trial at hand (useful for making population-level decisions) whereas cluster-specific effects allow consideration of the impact for a typical individual within a specific cluster. Exactly how to estimate a cluster-specific effect in the presence of informative clustering (and without being able to use a GLMM) remains unclear. In longitudinal designs, the target of inference also has a bearing on whether a cohort or cross-sectional design is more appropriate. If the unit of inference is the community, cross-sectional sampling might be best; if the unit of inference is the individual, cohort sampling might be best.^13^

## Implications

CRTs are used to evaluate a diverse range of interventions. Sometimes interest will be on the impact on the average individual, perhaps when evaluating a drug. In other settings, they might be used to evaluate an implementation strategy or an education package targeting healthcare providers. In these settings, we might be more interested in the impact of the intervention on the average cluster. The same trial may have several objectives linked to different outcomes and it might be the case that the target of inference differs for different outcomes. Even for the same outcome, we may want to draw inferences at both cluster and individual levels. Kahan’s paper underscores the need to clearly define the target of inference, which will involve discussions with trial investigators to determine whether interest is in the impact on the typical cluster or typical individual and then to select an analysis method that is likely to provide an unbiased estimate, irrespective of whether the cluster size is informative (as this will be mostly difficult to rule out).^4^ This is likely to make trials somewhat less efficient. More work is needed to address unanswered questions, such as methods of analysis for multiple period cluster trial designs, power considerations and methods for covariate adjustment particularly when using cluster-level approaches. Future reporting statements in cluster trials could be improved to require authors to clearly define their unit of inference and describe how they handled informative cluster sizes.

## Author contributions

Both authors made an equal contribution to the writing of the manuscript.

## Conflict of interest

None declared.
